# First person – Bao-Luen Chang

**DOI:** 10.1242/dmm.038182

**Published:** 2018-12-14

## Abstract

First Person is a series of interviews with the first authors of a selection of papers published in Disease Models & Mechanisms (DMM), helping early-career researchers promote themselves alongside their papers. Bao-Luen Chang is first author on ‘Semiology, clustering, periodicity and natural history of seizures in an experimental occipital epilepsy model’, published in DMM. Bao-Luen conducted the research described in this article while a PhD student in Professor Stephanie Schorge's lab at Department of Clinical and Experimental Epilepsy, UCL Queen Square Institute of Neurology (IoN), Queen Square, London, WC1N 3BG, UK. He is now a neurologist consultant and physician scientist in the lab of Professor Stephanie Schorge at No.5, Fuxing St., Guishan District, Taoyuan City 333, Taiwan, investigating mechanisms of epileptogenesis, epilepsy and pathophysiology of pharmacoresistant epilepsy.


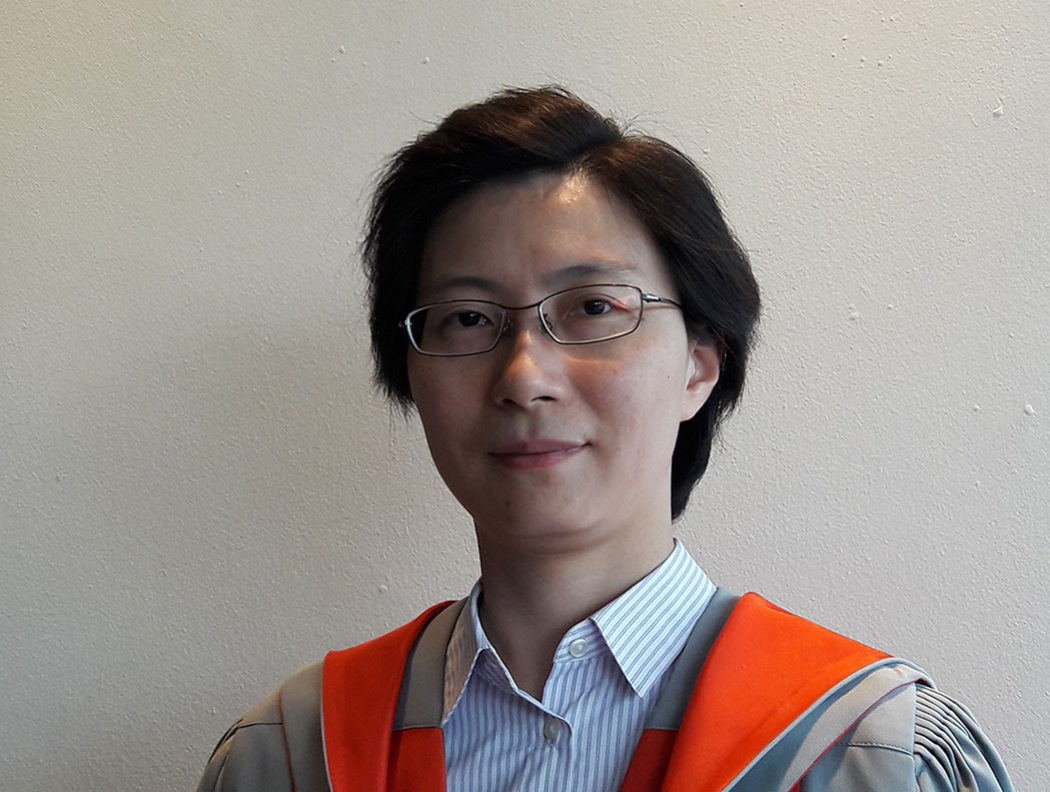


**Bao-Luen Chang**

**How would you explain the main findings of your paper to non-scientific family and friends?**

Epilepsy is a common and severe neurological disorder affecting over 50- to 60-million people worldwide. It is a lifelong brain disorder characterized by unprovoked and usually unpredictable seizures that seriously disrupt people's lives. Even with the best medicines available, 20-30% of people with epilepsy still have uncontrolled seizures. Of these people, approximately 75% have a type of epilepsy that starts in one place in the brain and spreads, called ‘focal epilepsy’, and seizures that start in tissue called ‘neocortical’ are the most likely to resist treatment by any medicines. For people with these types of seizures, the current best hope is that surgically removing the piece of the brain that causes seizures might stop the seizure but, for over 90% of people with focal epilepsy in neocortical tissue, the surgery is too risky, because removing the part of the brain that causes seizures might damage movement, language or vision. Most models of epilepsy in research represent a different type of epilepsy that arises from a part of the brain that can be removed, and consequently there is little known about how to treat the patients with focal neocortical epilepsy, and few studies looking at how this epilepsy may be different from the epilepsies that are currently treated by resection. Therefore, there is an urgent need to develop animal models that will allow scientists to test novel treatment strategies to help patients with this specific type of epilepsy, focal neocortical epilepsy. We generated a rat model of focal neocortical epilepsy and have provided detailed analysis of how it develops, and what the seizures look like. This model is designed to allow careful screening of treatments for the types of epilepsy that most need new medicines. We have shown that this model can be a valuable tool for studying the causes of epilepsy as well as for preclinical evaluation of new strategies to treat epilepsy.

**What are the potential implications of these results for your field of research?**

Currently, most of the commonly used animal models of epilepsy are modelled mesial temporal lobe epilepsy and triggered by initial status epilepticus (SE) accompanied by extensive neuronal death. Our rat model of occipital cortical epilepsy mimics focal neocortical epilepsy in humans, providing a non-SE- and non-lesion-induced epilepsy, and is a well-tolerated and reproducible model of epilepsy. Our results showed that this model exhibits a latent period followed by both short and long-lasting seizures, and it recapitulates many features of human neocortical epilepsy. Given the unmet clinical need for new therapies and the paucity of focal neocortical models of chronic epilepsy, this model is well suited to test for efficacy in developing new gene therapies and other novel treatment strategies, as well as for the study of epileptogenic mechanisms that lead to epilepsy.

**What are the main advantages and drawbacks of the model system you have used as it relates to the disease you are investigating?**

There are many types of epilepsy in humans, but most non-genetic animal models focus on a few types of temporal lobe epilepsy, meaning that patients with severe, pharmacoresistant focal neocortical epilepsy continue to have unmet needs. We have worked to develop a disease model to help address this gap in research. The major advantages of the tetanus toxin model of focal epilepsy are: the epileptogenesis is not triggered by SE and it does not rely upon neuronal loss; consequently, this model can be used to test focal treatments, which may be more appropriate than systemic drugs. Moreover, in this model there is a high induction rate of chronic epilepsy with very low morbidity and mortality, as well as a consistent latent period prior to the occurrence of spontaneous recurrent seizures. Importantly, this model has both clear electrographic ictal discharges and associated behavioral seizures that resemble human seizures and is an improvement compared to many other models that may only have epileptiform discharges without behavioral seizures. The drawback of this model is that, although robust seizures were observed, the seizure frequency could be highly variable among individual animals and gradually declined from 4-5 weeks after the onset of spontaneous seizures, leaving it unclear whether chronic epilepsy was fully established.

**Figure:**
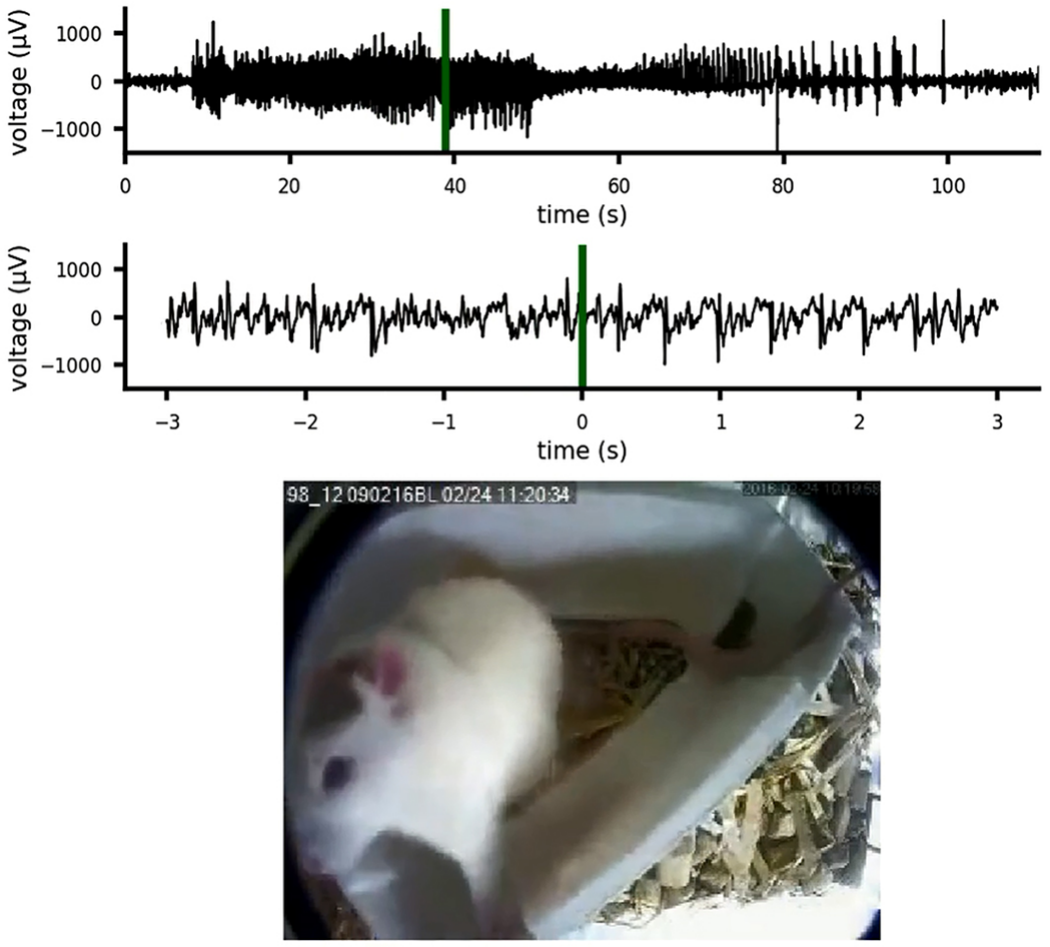
**A focal neocortical epilepsy model presenting as a focal seizure evolving to generalized tonic-clonic seizure.**

**What has surprised you the most while conducting your research?**

Seizure occurrence tended to cluster and also had a tendency for a periodic pattern. In addition, there is a positive correlation between the number of seizures in the first week and the remaining ∼4 weeks of recordings, and this is valuable as it allows a prediction algorithm to allocate animals into groups to provide similar seizure distribution in future tests of treatments. By grouping animals with similar seizure counts, the comparisons may be more robust with fewer animals, which is an important welfare consideration in these studies.

“The most significant challenge of epilepsy research is to develop pathway-driven discovery of novel therapeutic strategies.”

**Describe what you think is the most significant challenge impacting your research at this time and how will this be addressed over the next 10 years?**

The most significant challenge of epilepsy research is to develop pathway-driven discovery of novel therapeutic strategies. Since the mechanisms of epileptogenesis remain obscure and different epileptogenic mechanisms may be involved in different epilepsy syndromes, there are many opportunities that may yet be untested. On the other hand, different epileptic syndromes with diverse epileptogenic mechanisms may share some mechanisms of epileptogenesis, meaning that there may be opportunities to match treatments based on mechanisms. Applying appropriate disease models for seizures and epilepsies to understand the molecular signalling pathways during epileptogenesis, to dissect their spatiotemporal regulations and to seek the commonality among different epilepsy syndromes will greatly help us to develop treatments to cure and maybe even prevent epilepsy.

**What changes do you think could improve the professional lives of early-career scientists?**

Having the opportunity to learn and study scientific research at Professor Stephanie Schorge's lab in UCL Queen Square IoN has greatly improved my knowledge in basic neuroscience, which is invaluable for my future clinical and translational research. I am very fortunate to have Prof. Stephanie Schorge, Prof. Matthew C. Walker, and Prof. Dimitri M. Kullmann as my scientist mentors, who firmly articulate what science is and how a good scientist should work. From them, I have learned the objective attitude required to do fair scientific research and also to have a positive attitude to deal with issues between science and humanity; therefore, I have a clearer figure of how to be a good physician scientist in my career. By informing my understanding of the challenges and benefits of basic science, my early-career research will support my clinical research as well as my work with patients who have epilepsy.

“Hopefully, we can understand more about the mechanisms of epilepsy and eventually find translational therapeutic strategies that are not just symptomatic, but provide ‘anti-epileptogenesis’ and ‘disease-modifying treatment’ of epilepsy disorders.”

**What's next for you?**

As a neurologist consultant, I will make efforts to get balance between my clinical work and basic research on neuroscience, especially in the field of epilepsy. I will continue to investigate the mechanisms of the epileptogenic process and the pathophysiology of pharmacoresistance via both bench work and human study. Hopefully, we can understand more about the mechanisms of epilepsy and eventually find translational therapeutic strategies that are not just symptomatic, but provide ‘anti-epileptogenesis’ and ‘disease-modifying treatment’ of epilepsy disorders.
